# Diethyl *trans*-2,5-bis­(4-methoxy­benzyl­sulfan­yl)-1,4-dimethyl-3,6-dioxopiperazine-2,5-carboxyl­ate

**DOI:** 10.1107/S1600536809022211

**Published:** 2009-06-17

**Authors:** Nathan W. Polaske, Gary S. Nichol, Bogdan Olenyuk

**Affiliations:** a Department of Chemistry and Biochemistry, The University of Arizona, 1306 E. University Boulevard, Tucson, AZ 85721, USA

## Abstract

The title compound, C_28_H_34_N_2_O_8_S_2_, was synthesized as part of a project to develop synthetic routes to analogues of sporidesmins, a class of secondary metabolite produced by the filamentous fungi *Chaetomium* and *Pithomyces sp*. The complete molecule is generated by crystallographic inversion symmetry: the methoxy group is essentially coplanar with the benzene ring to which it is bonded, a mean plane fitted through the non-H atoms of the aromatic ring and the meth­oxy group having an r.m.s. deviation of 0.0140 Å. Similarly, the ester group is also essentially planar (r.m.s. deviation of a plane fitted through all non-H atoms is 0.0101 Å). There is only one independent C—H⋯O inter­action, which links together adjacent mol­ecules into a two-dimensional sheet in the *bc* plane.

## Related literature

For background information on the biological activity of sporidesmins, see: Fujimoto *et al.* (2004 ▶); Gardiner *et al.* (2005 ▶); Li *et al.* (2006 ▶); Saito *et al.* (1988 ▶); Waksman & Bugie (1944 ▶). For a discussion on the anti-cancer activity of these compounds, see: Brewer *et al.* (1978 ▶); Hauser *et al.* (1970 ▶); Kung *et al.* (2004 ▶); McInnes *et al.* (1976 ▶); Waksman & Bugie (1944 ▶). For related crystal structures, see: Isaka *et al.* (2005 ▶), Dubey *et al.* (2009 ▶); Polaske *et al.* (2009 ▶). For synthetic details, see: Hino & Sato (1974 ▶); Kawamura *et al.* (1975 ▶).
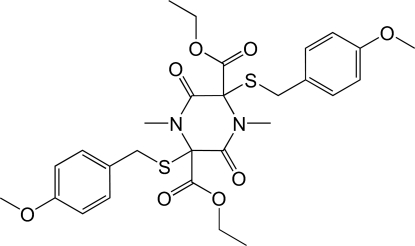


## Experimental

### 

#### Crystal data

C_28_H_34_N_2_O_8_S_2_
*M* *_r_* = 590.69
Monoclinic, 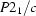

*a* = 11.290 (2) Å
*b* = 8.2259 (16) Å
*c* = 16.593 (3) Å
β = 109.704 (3)°
*V* = 1450.9 (5) Å^3^
*Z* = 2
Mo *K*α radiation
μ = 0.24 mm^−1^
*T* = 150 K
0.32 × 0.30 × 0.10 mm

#### Data collection

Bruker SMART 1000 CCD diffractometer
Absorption correction: multi-scan (*SADABS*; Sheldrick, 1996 ▶) *T*_min_ = 0.919, *T*_max_ = 0.987
11556 measured reflections
3522 independent reflections
2778 reflections with *I* > 2σ(*I*)
*R*_int_ = 0.028

#### Refinement

*R*[*F* ^2^ > 2σ(*F* ^2^)] = 0.036
*wR*(*F* ^2^) = 0.092
*S* = 1.03
3522 reflections
184 parameters
H-atom parameters constrained
Δρ_max_ = 0.38 e Å^−3^
Δρ_min_ = −0.24 e Å^−3^

### 

Data collection: *SMART* (Bruker, 2007 ▶); cell refinement: *SAINT* (Bruker, 2007 ▶); data reduction: *SAINT*; program(s) used to solve structure: *SHELXTL* (Sheldrick, 2008 ▶); program(s) used to refine structure: *SHELXTL*; molecular graphics: *ORTEP-3 for Windows* (Farrugia, 1997 ▶) and *Mercury* (Macrae *et al.*, 2008 ▶); software used to prepare material for publication: *SHELXTL*, *publCIF* (Westrip, 2009 ▶) and local programs.

## Supplementary Material

Crystal structure: contains datablocks I, global. DOI: 10.1107/S1600536809022211/fj2226sup1.cif

Structure factors: contains datablocks I. DOI: 10.1107/S1600536809022211/fj2226Isup2.hkl

Additional supplementary materials:  crystallographic information; 3D view; checkCIF report

## Figures and Tables

**Table 1:** Hydrogen-bond geometry (Å, °)

*D*—H⋯*A*	*D*—H	H⋯*A*	*D*⋯*A*	*D*—H⋯*A*
C5—H5⋯O4^i^	0.95	2.43	3.2647 (19)	147

Symmetry code: (i) 
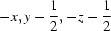
.

**Fig. 1.**
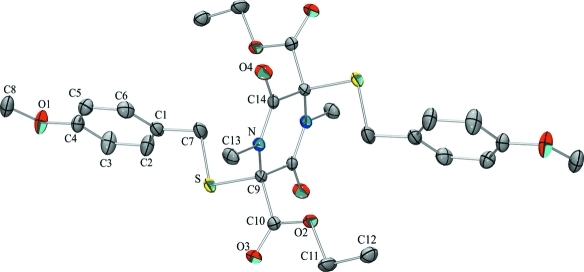
The molecular structure of (I). Displacement ellipsoids are at the 50% probability level and hydrogen atoms are omitted. Labelled atoms denote the asymmetric unit; unlabelled atoms are related by inversion symmetry (symmetry operator -x, -y + 1, -z).

**Fig. 2.**
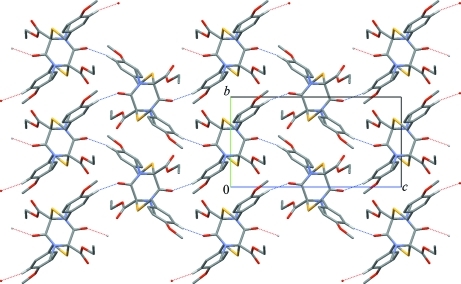
An a axis projection of the crystal packing of (I). Hydrogen bonding is indicated by dotted blue lines (dotted red lines indicate continuation of hydrogen bonding).

## supplementary crystallographic information

### Comment

Sporidesmins are an interesting class of secondary metabolites produced by the filamentous fungi *Chaetomium* and *Pithomyces sp*. This diverse class of natural products contains molecules with one or two epidithiodioxopiperazine rings that display a wide variety of biological activities (Waksman & Bugie, 1944; Saito *et al.*, 1988; Fujimoto *et al.*, 2004; Gardiner *et al.*, 2005; Li *et al.*, 2006). While toxic to mammalian cells, recent studies have suggested that certain sporidesmins may possess anticancer activity due to their ability to suppress neovascularization (Waksman & Bugie, 1944; Hauser *et al.*, 1970; McInnes *et al.*, 1976; Brewer *et al.*, 1978; Kung *et al.*, 2004). In the process of developing synthetic methodologies towards the synthesis of sporidesmin natural products, we came across a number of sulfenylated 2,5-piperazinediones whose structures could not be determined with confidence by NMR spectroscopy. Single crystal X-ray diffraction was found to be the only method available capable of unambiguously identifying the structures of these molecules. Herein we report the structure of the title compound (I).

The stucture of (I) is shown in Figure 1. Molecular dimensions are unexceptional and the compound crystallizes with crystallographic inversion symmetry in an extended conformation composed of essentially planar components. The methoxy group is essentially coplanar with the benzene ring to which it is bonded and a mean plane fitted through the non-hydrogen atoms of the aromatic ring and the methoxy group has an r.m.s. deviation of 0.0140 Å. Similarly the ester moiety is also essentially planar (r.m.s. deviation of a plane fitted through all non-hydrogen atoms is 0.0101 Å). The crystal packing has few notable intermolecular interactions; there is only one C–H···O interaction (plus an equivalent related by inversion symmetry) which links together adjacent molecules into a thick two-dimensional sheet in the *bc* plane.

### Experimental

To a dry flask equipped with a stir bar was added 1,4-dimethyl-3,6-diethoxycarbonyl-2,5-piperazinedione (Hino & Sato, 1974) (144 mg, 0.50 mmol), (DHQD)_2_PYR (88 mg, 0.10 mmol) and *N*-(4-methoxybenzylthio)succinimide (Kawamura *et al.*, 1975) (504 mg, 2.0 mmol). The compounds were then dried under vacuum for 15 minutes, followed by the addition of CH_2_Cl_2_ (2.25 ml). The mixture was allowed to stir at room temperature for 5 days. Once complete, the reaction was quenched with 1*M* KHSO_4_ (3 ml) and extracted with CH_2_Cl_2_ (3 *x* 15 ml). The organic extracts were combined, dried over anhydrous MgSO_4_, filtered, and concentrated under reduced pressure. Purification by column chromatography (silica gel, hexane:CH_2_Cl_2_:EtOAc (5:4:1)) followed by recrystallization from ethanol yielded colorless prisms (236 mg, 80% yield). LRMS (FAB, [*M*+H]+) found 591.36, C_28_H_35_N_2_O_8_S_2_ requires 591.18.

### Refinement

Hydrogen atoms were identified from a difference map and refined with *U*_iso_(H)= 1.5*U*_eq_(C) (methyl H atoms) and *U*_iso_(H)= 1.2*U*_eq_(C) for all others. Fixed C–H distances of 0.95Å (aryl), 0.98Å (methyl) and 0.99Å (methylene) were used.

### Crystal data

**Table:**

C_28_H_34_N_2_O_8_S_2_	*F*(000) = 624
*M**_r_* = 590.69	*D*_x_ = 1.352 Mg m^−^^3^
Monoclinic, *P*2_1_/*c*	Mo *K*α radiation, λ = 0.71073 Å
Hall symbol: -P 2ybc	Cell parameters from 5275 reflections
*a* = 11.290 (2) Å	θ = 2.3–28.3°
*b* = 8.2259 (16) Å	µ = 0.24 mm^−^^1^
*c* = 16.593 (3) Å	*T* = 150 K
β = 109.704 (3)°	Plate, colourless
*V* = 1450.9 (5) Å^3^	0.32 × 0.30 × 0.10 mm
*Z* = 2	

### Data collection

**Table:**

Bruker SMART 1000 CCD diffractometer	3522 independent reflections
Radiation source: sealed tube	2778 reflections with *I* > 2σ(*I*)
graphite	*R*_int_ = 0.028
Thin–slice ω scans	θ_max_ = 28.3°, θ_min_ = 1.9°
Absorption correction: multi-scan (*SADABS*; Sheldrick, 1996)	*h* = −14→14
*T*_min_ = 0.919, *T*_max_ = 0.987	*k* = −10→10
11556 measured reflections	*l* = −21→22

### Refinement

**Table:**

Refinement on *F*^2^	Primary atom site location: structure-invariant direct methods
Least-squares matrix: full	Secondary atom site location: difference Fourier map
*R*[*F*^2^ > 2σ(*F*^2^)] = 0.036	Hydrogen site location: difference Fourier map
*wR*(*F*^2^) = 0.092	H-atom parameters constrained
*S* = 1.03	*w* = 1/[σ^2^(*F*_o_^2^) + (0.0397*P*)^2^ + 0.678*P*] where *P* = (*F*_o_^2^ + 2*F*_c_^2^)/3
3522 reflections	(Δ/σ)_max_ < 0.001
184 parameters	Δρ_max_ = 0.38 e Å^−^^3^
0 restraints	Δρ_min_ = −0.24 e Å^−^^3^

### Special details

**Table:**

Geometry. All e.s.d.'s (except the e.s.d. in the dihedral angle between two l.s. planes) are estimated using the full covariance matrix. The cell e.s.d.'s are taken into account individually in the estimation of e.s.d.'s in distances, angles and torsion angles; correlations between e.s.d.'s in cell parameters are only used when they are defined by crystal symmetry. An approximate (isotropic) treatment of cell e.s.d.'s is used for estimating e.s.d.'s involving l.s. planes.
Refinement. Refinement of *F*^2^ against ALL reflections. The weighted *R*-factor *wR* and goodness of fit *S* are based on *F*^2^, conventional *R*-factors *R* are based on *F*, with *F* set to zero for negative *F*^2^. The threshold expression of *F*^2^ > σ(*F*^2^) is used only for calculating *R*-factors(gt) *etc*. and is not relevant to the choice of reflections for refinement. *R*-factors based on *F*^2^ are statistically about twice as large as those based on *F*, and *R*- factors based on ALL data will be even larger.

### Fractional atomic coordinates and isotropic or equivalent isotropic displacement parameters (Å^2^)

**Table:**

	*x*	*y*	*z*	*U*_iso_*/*U*_eq_	
S	0.18848 (3)	0.24066 (4)	0.04288 (2)	0.02157 (10)	
O1	0.59043 (11)	0.03836 (16)	−0.14348 (8)	0.0357 (3)	
O2	−0.08416 (10)	0.32864 (14)	0.13178 (7)	0.0271 (3)	
O3	0.07865 (11)	0.15503 (14)	0.16459 (7)	0.0301 (3)	
O4	−0.16577 (11)	0.45732 (14)	−0.15608 (7)	0.0283 (3)	
N	−0.05515 (11)	0.34842 (14)	−0.02822 (7)	0.0168 (2)	
C1	0.32030 (15)	0.27272 (19)	−0.06556 (10)	0.0251 (3)	
C2	0.44789 (16)	0.2787 (2)	−0.01800 (11)	0.0336 (4)	
H2	0.4754	0.3387	0.0339	0.040*	
C3	0.53537 (16)	0.1986 (2)	−0.04519 (12)	0.0363 (4)	
H3	0.6222	0.2038	−0.0118	0.044*	
C4	0.49678 (14)	0.1106 (2)	−0.12108 (10)	0.0247 (3)	
C5	0.37024 (14)	0.1019 (2)	−0.16888 (10)	0.0256 (3)	
H5	0.3426	0.0411	−0.2206	0.031*	
C6	0.28382 (15)	0.1832 (2)	−0.14039 (11)	0.0283 (4)	
H6	0.1970	0.1770	−0.1734	0.034*	
C7	0.22463 (17)	0.3623 (2)	−0.03733 (12)	0.0313 (4)	
H7A	0.1472	0.3810	−0.0870	0.038*	
H7B	0.2587	0.4692	−0.0129	0.038*	
C8	0.55425 (18)	−0.0657 (2)	−0.21608 (12)	0.0361 (4)	
H8A	0.5000	−0.1524	−0.2077	0.054*	
H8B	0.6295	−0.1137	−0.2233	0.054*	
H8C	0.5084	−0.0029	−0.2673	0.054*	
C9	0.05188 (13)	0.35374 (17)	0.05104 (9)	0.0167 (3)	
C10	0.01900 (14)	0.26530 (18)	0.12339 (9)	0.0201 (3)	
C11	−0.12546 (17)	0.2576 (2)	0.19906 (11)	0.0353 (4)	
H11A	−0.0596	0.2720	0.2557	0.042*	
H11B	−0.1417	0.1399	0.1887	0.042*	
C12	−0.24275 (16)	0.3430 (2)	0.19672 (11)	0.0304 (4)	
H12A	−0.2258	0.4595	0.2063	0.046*	
H12B	−0.2721	0.2993	0.2416	0.046*	
H12C	−0.3077	0.3263	0.1407	0.046*	
C13	−0.10631 (15)	0.18707 (19)	−0.05932 (10)	0.0258 (3)	
H13A	−0.1961	0.1967	−0.0923	0.039*	
H13B	−0.0950	0.1145	−0.0104	0.039*	
H13C	−0.0619	0.1424	−0.0959	0.039*	
C14	−0.09368 (13)	0.47399 (17)	−0.08292 (9)	0.0178 (3)	

### Atomic displacement parameters (Å^2^)

**Table:**

	*U*^11^	*U*^22^	*U*^33^	*U*^12^	*U*^13^	*U*^23^
S	0.02166 (18)	0.01810 (18)	0.02652 (19)	0.00695 (14)	0.01018 (14)	0.00412 (15)
O1	0.0249 (6)	0.0441 (7)	0.0408 (7)	0.0008 (5)	0.0145 (5)	−0.0176 (6)
O2	0.0304 (6)	0.0287 (6)	0.0284 (6)	0.0097 (5)	0.0184 (5)	0.0123 (5)
O3	0.0366 (6)	0.0273 (6)	0.0291 (6)	0.0119 (5)	0.0147 (5)	0.0126 (5)
O4	0.0332 (6)	0.0242 (6)	0.0189 (5)	0.0013 (5)	−0.0027 (5)	−0.0015 (4)
N	0.0183 (6)	0.0145 (6)	0.0161 (6)	0.0000 (4)	0.0037 (5)	−0.0009 (5)
C1	0.0254 (8)	0.0225 (8)	0.0316 (8)	0.0054 (6)	0.0152 (7)	0.0069 (6)
C2	0.0301 (9)	0.0408 (10)	0.0309 (9)	0.0015 (7)	0.0117 (7)	−0.0124 (8)
C3	0.0183 (8)	0.0505 (11)	0.0367 (10)	0.0005 (7)	0.0047 (7)	−0.0152 (8)
C4	0.0211 (7)	0.0271 (8)	0.0283 (8)	0.0015 (6)	0.0116 (6)	−0.0024 (7)
C5	0.0237 (8)	0.0287 (8)	0.0237 (8)	−0.0021 (6)	0.0069 (6)	−0.0034 (6)
C6	0.0178 (7)	0.0342 (9)	0.0311 (8)	0.0026 (6)	0.0059 (6)	0.0052 (7)
C7	0.0341 (9)	0.0250 (8)	0.0439 (10)	0.0109 (7)	0.0252 (8)	0.0104 (7)
C8	0.0418 (10)	0.0350 (10)	0.0387 (10)	−0.0007 (8)	0.0228 (8)	−0.0108 (8)
C9	0.0173 (7)	0.0161 (7)	0.0158 (6)	0.0028 (5)	0.0046 (5)	0.0013 (5)
C10	0.0237 (7)	0.0191 (7)	0.0175 (7)	0.0013 (6)	0.0070 (6)	0.0006 (6)
C11	0.0384 (10)	0.0417 (10)	0.0341 (9)	0.0085 (8)	0.0233 (8)	0.0183 (8)
C12	0.0295 (8)	0.0381 (10)	0.0272 (8)	0.0022 (7)	0.0141 (7)	0.0070 (7)
C13	0.0292 (8)	0.0166 (7)	0.0273 (8)	−0.0032 (6)	0.0038 (7)	−0.0009 (6)
C14	0.0180 (7)	0.0178 (7)	0.0180 (7)	0.0024 (5)	0.0066 (6)	−0.0017 (5)

### Geometric parameters (Å, °)

**Table:**

S—C7	1.8181 (17)	C5—C6	1.391 (2)
S—C9	1.8447 (14)	C6—H6	0.9500
O1—C4	1.3689 (19)	C7—H7A	0.9900
O1—C8	1.421 (2)	C7—H7B	0.9900
O2—C10	1.3255 (18)	C8—H8A	0.9800
O2—C11	1.4682 (19)	C8—H8B	0.9800
O3—C10	1.1972 (18)	C8—H8C	0.9800
O4—C14	1.2201 (17)	C9—C10	1.552 (2)
N—C9	1.4560 (17)	C9—C14^i^	1.531 (2)
N—C13	1.4698 (19)	C11—H11A	0.9900
N—C14	1.3469 (18)	C11—H11B	0.9900
C1—C2	1.391 (2)	C11—C12	1.488 (2)
C1—C6	1.382 (2)	C12—H12A	0.9800
C1—C7	1.507 (2)	C12—H12B	0.9800
C2—H2	0.9500	C12—H12C	0.9800
C2—C3	1.383 (2)	C13—H13A	0.9800
C3—H3	0.9500	C13—H13B	0.9800
C3—C4	1.389 (2)	C13—H13C	0.9800
C4—C5	1.382 (2)	C14—C9^i^	1.531 (2)
C5—H5	0.9500		
			
C7—S—C9	100.12 (7)	H8A—C8—H8B	109.5
C4—O1—C8	117.65 (13)	H8A—C8—H8C	109.5
C10—O2—C11	116.04 (12)	H8B—C8—H8C	109.5
C9—N—C13	116.90 (11)	S—C9—N	112.23 (9)
C9—N—C14	124.53 (12)	S—C9—C10	104.14 (9)
C13—N—C14	117.16 (12)	S—C9—C14^i^	108.92 (9)
C2—C1—C6	117.82 (15)	N—C9—C10	110.02 (11)
C2—C1—C7	121.36 (16)	N—C9—C14^i^	113.91 (11)
C6—C1—C7	120.82 (15)	C10—C9—C14^i^	107.03 (11)
C1—C2—H2	119.5	O2—C10—O3	125.65 (14)
C1—C2—C3	121.00 (16)	O2—C10—C9	110.17 (12)
H2—C2—C3	119.5	O3—C10—C9	124.17 (13)
C2—C3—H3	119.9	O2—C11—H11A	110.2
C2—C3—C4	120.24 (15)	O2—C11—H11B	110.2
H3—C3—C4	119.9	O2—C11—C12	107.49 (13)
O1—C4—C3	115.94 (14)	H11A—C11—H11B	108.5
O1—C4—C5	124.42 (14)	H11A—C11—C12	110.2
C3—C4—C5	119.64 (15)	H11B—C11—C12	110.2
C4—C5—H5	120.4	C11—C12—H12A	109.5
C4—C5—C6	119.20 (15)	C11—C12—H12B	109.5
H5—C5—C6	120.4	C11—C12—H12C	109.5
C1—C6—C5	122.08 (14)	H12A—C12—H12B	109.5
C1—C6—H6	119.0	H12A—C12—H12C	109.5
C5—C6—H6	119.0	H12B—C12—H12C	109.5
S—C7—C1	108.65 (11)	N—C13—H13A	109.5
S—C7—H7A	110.0	N—C13—H13B	109.5
S—C7—H7B	110.0	N—C13—H13C	109.5
C1—C7—H7A	110.0	H13A—C13—H13B	109.5
C1—C7—H7B	110.0	H13A—C13—H13C	109.5
H7A—C7—H7B	108.3	H13B—C13—H13C	109.5
O1—C8—H8A	109.5	O4—C14—N	122.68 (13)
O1—C8—H8B	109.5	O4—C14—C9^i^	118.20 (13)
O1—C8—H8C	109.5	N—C14—C9^i^	119.02 (12)
			
C6—C1—C2—C3	0.5 (3)	C14—N—C9—C10	138.48 (13)
C7—C1—C2—C3	−178.77 (17)	C14—N—C9—C14^i^	18.3 (2)
C1—C2—C3—C4	0.1 (3)	C7—S—C9—N	64.78 (12)
C8—O1—C4—C3	173.97 (17)	C7—S—C9—C10	−176.24 (10)
C8—O1—C4—C5	−6.6 (2)	C7—S—C9—C14^i^	−62.31 (11)
C2—C3—C4—O1	178.72 (17)	C11—O2—C10—O3	1.3 (2)
C2—C3—C4—C5	−0.7 (3)	C11—O2—C10—C9	−179.23 (13)
O1—C4—C5—C6	−178.71 (16)	S—C9—C10—O2	−175.74 (10)
C3—C4—C5—C6	0.7 (3)	S—C9—C10—O3	3.69 (18)
C2—C1—C6—C5	−0.5 (2)	N—C9—C10—O2	−55.27 (15)
C7—C1—C6—C5	178.73 (15)	N—C9—C10—O3	124.16 (15)
C4—C5—C6—C1	0.0 (3)	C14^i^—C9—C10—O2	68.99 (14)
C2—C1—C7—S	−81.68 (18)	C14^i^—C9—C10—O3	−111.58 (16)
C6—C1—C7—S	99.06 (17)	C10—O2—C11—C12	−178.98 (14)
C9—S—C7—C1	−169.77 (12)	C9—N—C14—O4	164.45 (14)
C13—N—C9—S	59.98 (15)	C9—N—C14—C9^i^	−19.2 (2)
C13—N—C9—C10	−55.48 (16)	C13—N—C14—O4	−1.6 (2)
C13—N—C9—C14^i^	−175.65 (12)	C13—N—C14—C9^i^	174.82 (12)
C14—N—C9—S	−106.05 (13)		

Symmetry codes: (i) −*x*, −*y*+1, −*z*.

### Hydrogen-bond geometry (Å, °)

**Table:**

*D*—H···*A*	*D*—H	H···*A*	*D*···*A*	*D*—H···*A*
C5—H5···O4^ii^	0.95	2.43	3.2647 (19)	147

Symmetry codes: (ii) −*x*, *y*−1/2, −*z*−1/2.
